# Crystal Structure of Fad35R from *Mycobacterium tuberculosis* H37Rv in the Apo-State

**DOI:** 10.1371/journal.pone.0124333

**Published:** 2015-05-04

**Authors:** Appu Kumar Singh, Babu Manjasetty, Balasubramani GL, Sukirte Koul, Abhishek Kaushik, Mary Krishna Ekka, Vijay Singh, S. Kumaran

**Affiliations:** 1 Council of Scientific and Industrial Research (CSIR), Institute of Microbial Technology, G.N.Ramachandran Protein Centre, Chandigarh, 160036, India; 2 European Molecular Biology Laboratory, Grenoble Outstation, 38000, France; 3 Unit for Virus Host-Cell Interactions, Univ. Grenoble Alpes-EMBL-CNRS, 38000, France; University of South Florida College of Medicine, UNITED STATES

## Abstract

Fad35R from *Mycobacterium tuberculosis* binds to the promoter site of Fad35 operon and its DNA binding activities are reduced in the presence of tetracycline and palmitoyl-CoA. We resolved the crystal structure of Fad35R using single-wavelength anomalous diffraction method (SAD). Fad35R comprises canonical DNA binding domain (DBD) and ligand binding domain (LBD), but displays several distinct structural features. Two recognition helices of two monomers in the homodimer are separated by ~ 48 Å and two core triangle-shaped ligand binding cavities are well exposed to solvent. Structural comparison with DesT and QacR structures suggests that ligand binding-induced movement of α7, which adopts a straight conformation in the Fad35R, may be crucial to switch the conformational states between repressive and derepressive forms. Two DBDs are packed asymmetrically, creating an alternative dimer interface which coincides with the possible tetramer interface that connects the two canonical dimers. Quaternary state of alternative dimer mimics a closed-state structure in which two recognition helices are distanced at ~ 35 Å and ligand binding pockets are inaccessible. Results of biophysical studies indicate that Fad35R has the propensity to oligomerize in solution in the presence of tetracycline. We present the first structure of a FadR homologue from mycobacterium and the structure reveals DNA and ligand binding features of Fad35R and also provides a view on alternative quaternary states that mimic open and closed forms of the regulator.

## Introduction

*Mycobacterium tuberculosis (M*. *tuberculosis)* encodes an unusually large number of genes involved in fatty acids (FA) metabolism [1]. Tight control over fatty acid homeostasis is important for growth, survival, and pathogenicity of *M*. *tuberculosis* [2]. Therefore, understanding the regulatory mechanisms that govern FA biosynthesis and degradation in *M*. *tuberculosis* are an active area of research [3–5]. Transcriptional regulation is one of the major mechanism by which expression of genes are controlled in response to changing conditions. More than 200 genes, 6% of the *M*. *tuberculosis* genome are predicted to be dedicated to FA metabolism [1]. Among these, ~ 100 were predicted to function in β-oxidation of FA in *M*. *tuberculosis* whereas *E*. *coli* which encodes only one enzyme for this function [1,6]. Bioinformatics analysis shows that *M*. *smegmatis* genome encodes a large number of transcriptional regulators (~ 150–200) and, 25–30% of them are predicted to function as FadRs, a type of transcription factors that are known to regulate the expression of genes involved in FA metabolism [7,8]. Since *M*. *smegmatis* and *M*. *tuberculosis* genomes display similar genetic architectures, *M*. *tuberculosis* may also possess similar number of FadR genes. A large number of enzymes and transcription factors dedicated to FA metabolism and its regulation, reflects the complexity of lipid metabolism in mycobacterium. However, only few FadR genes from mycobacterium have been characterized so far and no crystal structure of FadR homologues from mycobacterium has been resolved to date [9,10].

We recently characterized the promoter binding properties of Fad35R, a FadR homologue from *M*. *tuberculosis* [9]. Fad35R has been annotated as a member of TTR family [1]. Our recent study showed that DNA binding activities of Fad35R are sensitive to tetracycline as well as to long chain FA. We proposed that Fad35R might be one of regulators of the FA metabolism in mycobacterium [10]. Recent studies classify FadR homologues in to GntR family and therefore, Fad35R may be considered as a member of GntR family [8]. Fad35R shows very low sequence identity (< 18%) with other known FadR homologues and in addition, it consists of a 23-residue N-terminal extension, not present in other FadR homologues Our study showed that Fad35R binds strongly to a promoter site located upstream of the Fad35 gene (acyl-CoA synthase). Fad35 gene is located next to the ScoA-CitE operon which encodes a group of six enzymes that carry out the first committed acetyl-CoA generating step of ketone bodies degradation, fatty acid degradation, and citric acid cycle. Activated fatty acids and citrate are metabolites of Fad35 and ScoA-CitE operon and also, they act as regulatory ligands of Fad35R by changing its DNA binding affinity [10]. Therefore, Fad35R and metabolites of Sco-CitE operon form a transcriptional circuit that would sense time dependent variations in metabolite concentrations and control Fad35R activity accordingly. Information on structural and biochemical signatures of Fad35R and its interactions towards ligands and DNA would allow us to capture the key regulatory features of the transcriptional circuit. Using sequence comparison and homology modeling approaches, we showed that Fad35R consists of a canonical N-terminal Helix-Turn-Helix (HTH) containing DNA binding domain (DBD) and a C-terminal ligand binding domain (LBD) [10]. However, modeling studies could not be extended to predict structural features of DNA and ligand binding domains. Due to high sequence diversity, structural features of LBDs of TTR members are known to vary significantly [11].

The canonical derepression mechanism of TTRs involves a series of steps which result in the dissociation of the regulator from the DNA, upon ligand binding. It is well known that the binding of an inducer ligand to the LBD triggers allosteric conformational changes which propagate to the DBD and modulate its DNA binding properties by altering its conformation[11,12]. Despite the conservation of derepression mechanism, DNA recognition modes of TTR members differ significantly due to the inherent structural differences between members [13–16]. For example, QacR binds cooperatively to the 28 bp palindrome as tetramer without bending the DNA much whereas TetR binds to a 15 bp palindrome as dimer and bends the binding site upon binding [13,16]. Other notable difference is the existence of two different quaternary states of SCO0520, a TTR family regulator from *Streptomyces coelicolor* (PDB: 2Q24) and the propensity of SCO0520 oligomerize in solution [17]. Different DNA binding modes may also be influenced by different ligands which bind to the same ligand binding cavity of LBD. Recent crystal structures of DesT in complex with palmitoyl-CoA or oleoyl-CoA showed that tails of both FA bind deep within the hydrophobic ligand binding cavity of DesT [18]. However, binding modes of palmitoyl-CoA and oleoyl-CoA are different and they exert opposite effects on the DNA binding properties of DesT. Upon binding, each FA induces a different set of conformational changes that dictate the structural and functional status of the protein either to be in "repressive" or "de-repressive" state. Like DesT, Fad35R binds to the promoter region of Fad35 gene which consists of two 8 bp direct repeats, connected by a 8bp linker. The study showed that promoter binding activities of Fad35R are modulated in the presence of palmitoyl-CoA. In addition, Fad35R also binds other ligands such as tetracycline and citrate, suggesting that Fad35R binds a variety of structurally diverse ligands [10]. To aid further understanding of Fad35R mediated transcriptional regulation in *mycobacterium*, we resolved the crystal structure of Fad35R from *M*. *tuberculosis* using SAD phasing and examined its unique structural features.

Here, the crystal structure of Fad35R, the first structure of FadR homologue from *M*. *tuberculosis*, is resolved at 3.4 Å resolution. Fad35R consists of a canonical TTR family fold with a small DNA binding domain at the N-terminus and a larger ligand binding domain at the C- terminus. We report that the ligand-free form of Fad35R forms two distinct types of dimers. The canonical dimer mimics the "open" state and the non-canonical dimer represents a "closed" state. Alternative and crystallographic dimers of symmetry mates generate higher-order oligomeric structure. We describe the unique structural and regulatory features of Fad35R here.

## Materials and Methods

### Materials

All chemicals and reagents were of analytical reagent grade and were procured from different commercial sources. All oligonucleotides used in this study were of analytical quality and obtained either from Midland certified reagent company (U.S.A) or Sigma (U.S.A).

### Protein expression and purification

Cloning, expression, and purification of native and Selenomethionine containing Fad35R (Fad35R-SeMet) were performed as described previously [10,19]. Ni-NTA affinity chromatography purified protein was subjected to thrombin cleavage, passed through Ni-NTA column. Affinity purified protein was further purified by gel-filtration chromatography on Hiprep 26/60 Sephacryl S-200 column. The purity of Fad35R was monitored in 10% SDS-PAGE gel followed by Coomassie brilliant staining and the purity was found to be > 98%.

### Crystallization, structure determination and refinement

Size-exclusion purified Fad35R-SeMet was used for crystallization. We screened for crystallization conditions using Nextel and ammonium sulphate screening suites (Nextal Classic suite-96, Qiagen Sciences, Maryland USA). Using the sitting drop method, 1μL of protein (30 mg/mL) stock was mixed with 1μL of reservoir solution and the mixture was equilibrated against 80 μL of precipitant solution. Diffraction quality Fad35R-SeMet crystals grew in two weeks and the optimum crystallization condition was found to be the following; 0.2 M Tris-sodium citrate, 0.1 M Tris-HCl, pH 8.5, 30% (v/v) PEG400. We used single wavelength anomalous dispersion (SAD) phasing method for resolving the structure. Fad35R-SeMet crystals diffracted to 3.4 Å and the SAD dataset was obtained at Se edge (λ = 0.9775Å) on a MARCCD 225 detector at the BM14 beamline of the European Synchrotron facility (ESRF, France). The oscillation angle was kept as 1°, the exposure time was 40 sec per frame, and the detector distance was 385 mm. The peak dataset were then indexed, integrated, and scaled using the HKL2000 suite [20]. Data collection statistics are shown in Table 1. Each monomer of Fad35R consists of three SeMet residues and the positions of the Se atoms were determined used SHELXD program (correlation coefficient, CC all/ weak: 53.81/27.55; Patterson figure of merit, PATFOM 15.01) [21]. The initial phases were computed and partial model was built with SHELXE as part of the HKL2MAP package [22,23]. The resulting phases were of poor quality and the electron density was not continuous to trace the entire molecule. However, the model quality was improved by the iterative manual model building using COOT and followed by model refinement using the REFMAC [24,25]. The model was further improved by several rounds of SAD phasing using PHASER [26], phase combination using SIGMAA [27] density modification using PIRATE [28] and iterative model building with BUCCANEER [29]. At this stage, the phases were improved and electron density map was continuous to trace the missing residues in the model except for the N-terminal residues, 1 to 23. The automatic model building was not successful in building the entire molecule due to the limitation of resolution. The remaining part of the structure, including side chains were modeled manually. The model was refined using the program REFMAC5 and iterative manual rebuilding of the model is performed in COOT. Two glycerol molecules were identified and included in the refinement. The Translation, Libration and Screw-rotation (TLS) displacement parameters were determined for the one group for each molecule and TLS-restrained refinement was performed [30]. For the final model, the *R*_work_ is 20.8% and *R*_free_ is 25.6%. The structure has good stereochemistry as indicated by program PROCHECK [31] and final refinement statistics are shown in Table 1. The refined model of Fad35R and structure factors were deposited in the Protein Data Bank under the accession code 4G12. Surface area was calculated with the PDBe PISA server [32].

**Table 1:** Data collection and refinement statistics.

Dataset	Fad35R
**Data collection** ^***a***^	
Wavelength	0.9775
Space group	P2_1_2_1_2_1_
Unit cell dimensions	
*a*, *b*, *c* (Å)	76.84, 83.21, 95.33
*α = β = γ* (deg)	90
Resolutions (Å)	50–3.44 (3.56–3.43)
Total Reflections	67789
Unique Reflections	15712 (818)
Completeness (%)^***a***^ ^,^ ^b^	95
Redundancy	2.9 (2.0)
R_merge_ (%)^b^ ^,^ ^c^	12.2 (52.9)
I/σ_*I*_	12.4 (2.54)
ShelxD^d^: Data used (Å)	4
Correlation coffefficient CC) all/weak	53.81/27.55
Combined figure of merit (CFOM)	81.4
PAT figure of merit (FOM)	15.01
**Refinement**	
No. of reflections, work/free	7538/944
R_work_ (%)^***a***^	20.8 (27.3)
R_free_ (%)	25.6 (38.58)
Root-mean-square deviation for bonds (Å)	0.005
Root-mean-square deviation for angle (deg)	0.862
No. of protein molecules	2
No. of atoms in protein molecules	2851
Ligand	12
Water	0
Average *B* factor (Å^2^)	75.10
**Ramachandran plot (%)**	
Favored regions	91.2
Allowed regions	8.8
Generously allowed regions	0.0
Outliers	0.0
PDB entry	4G12

^*a*^ *Data completeness treats Bijvoët mates independently*.

^*b*^ *Statistics for the highest resolution bin are given in parentheses*.

^*c*^ *R*_*merge*_ = *∑*_*hkl*_ *∑*_*i*_ *|I(hkl)*_*i*_ *|−< I(hkl) >|/∑*_*hkl*_ *∑*_*i*_ *< I(hkl)*_*i*_ *>*.

^*d*^ *Substructure determination parameters are from ShelxD*.

^*a*^ *R*_*work*_ = *∑*_*hkl*_ *||F*_*o*_ *(hkl)|−k|F*_*c*_ *(hkl)||/∑*_*hkl*_ *|F*_*o*_ *(hkl)|*, *where F*_*o*_ *and F*_*c*_ *are observed and calculated structure factors*.

### Dynamic light scattering studies

Dynamic light scattering studies were performed using Beckman Coulter (delsa Nanose) instruments equipped with 512 channels. He-Ne laser used with 45 mwatt power and angle of scattering was 165°. Measurements were performed with 100 μm aperture using glass cuvette containing 1.8 mL of sample in a cell holder equipped with a thermostat. The autocorrelation function of the scattering data was analyzed using the following equation to obtain the translation diffusion coefficient of the protein.

$$G(t)=B+A\;\text{exp}(-\frac{t}{\tau })$$ (eq 1)

Where τ is the auto correlation time and it is defined as

$$\tau =\frac{1}{2Dq^{2}}$$ (eq 2)

Where D is the diffusion coefficient and q is the scattering vector.

### Sedimentation velocity

Sedimentation velocity experiments were performed using an Optima XL-I analytical ultracentrifuge equipped with absorbance optics with an An50Ti rotor (Beckman Coulter). Gel filtration purified Fad35R was extensively dialyzed in the standard buffer (50 mM Tris-Cl, pH 8.0, 300 mM NaCl, 10% glycerol, 0.1 mM DTT) and protein filtered with 0.22 μM was used. Sedimentation velocity studies were carried out at 45,000 rpm at 20°C using a two-channel charcoal-filled centerpiece with sapphire windows. Velocity data were collected by scanning samples at a wavelength of 280 nm with a spacing of 0.003 cm and an average of 3 scans per step. The partial specific volume, solvent density and viscosity were calculated using SEDNTERP [33]. The molecular weight for Fad35R was obtained by fitting the sedimentation data with Sedfit, freely available analysis software [34].

## Results

### Structure determination and quality of the structure

**Fig 1:**
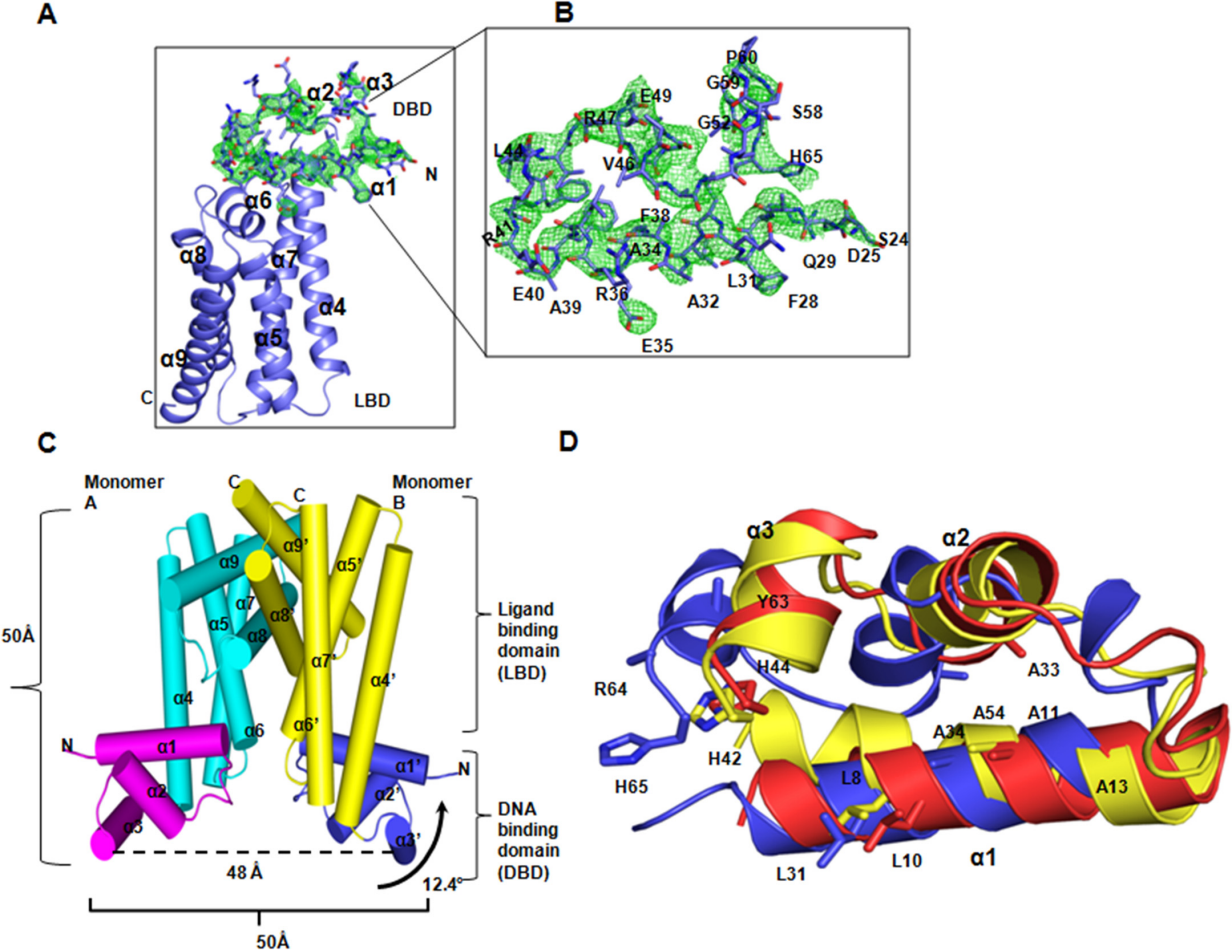
Structural description of Fad35R. A) A monomer of Fad35R, shown in blue color; the ligand binding domain shown in cartoon and the DNA binding domain shown as sticks, superimposed with Fo-Fc map (green). B) Close-up view of Fo-Fc map (green) and corresponding residues of DBD. The Fo-Fc is contoured at 2.5σ. C) Overview of Fad35R homodimer. Dimer is shown as cylindrical representation and respective helices of two monomers (cyan and yellow) are labeled as α and α'. The dimension of the dimer is indicated and distance between two recognition helices, α3 and α3' is shown at the bottom. The twisting of one the DBD around the two-fold NCS axis is also shown. D) Superimposition of DBDs of QacR (yellow), CgmR (red) with that of Fad35R (blue). The conserved and DNA binding residues are shown sticks.

Fad35R consists of 215 amino acid residues (Genbank accession number CAB08924.1). The BLAST search reveals that the protein has conserved domains of the TTRs but overall sequence identity is low (~ 18%). Therefore, we determined the crystal structure of Fad35R by the SAD method, and refined it to 3.4 Å resolution. The final model of Fad35R comprises of two monomers in the asymmetric unit, containing 384 residues in *P*2_1_2_1_2_1_space group with unit cell dimension of a = 76.8, b = 83.2, c = 95.3. The final model of Fad35R is of good quality as indicated by low gap between the R_work_ (21.3%) and R_free_ (25.5%) and good RMSD from ideal bond angle (0.817 Å) and bond length (0.005 Å). Stereochemistry of the Fad35R, checked by PROCHECK indicate that 91% residues are in most favorable region and the rest fall in the additionally allowed region of the Ramachandran plot (**Table 1**). With the exception of the N-terminal residues 1–24, for which no electron density is observed, the polypeptide chain of each monomer can be unambiguously traced from the N-terminus to C-terminus (**Fig 1A and 1B**). The disordered first 24 residues of N-terminal which immediately precede the conserved DNA binding helix-turn-helix (HTH) motif may likely to be ordered upon DNA binding as observed in the structure of SimR [15]. During refinement, unaccounted electron density was observed at the predicted ligand binding pocket. Since the chemical identity of the ligand was not known and no known ligand was mixed with the protein prior to crystallization, we modeled a glycerol moiety into the density as it was present in purification buffer as well as in the cryoprotectant solution.

### Overall Structure of Fad35R

The asymmetric unit of the crystal comprised of one homodimer (chain A and B) which adopts a ‘Ω’ (Ohm) shaped canonical TTR family fold with dimensions of 50Å x 48Å x 35Å (**Fig 1C**). The overall structure of Fad35R monomer includes a total of 192 amino acids, folded into nine alpha helices (α1-α9) which are dispersed into two domains; a smaller DNA-binding N-terminal domain (DBD: S^24^-E^70^, α1-α3) and a larger ligand-binding C-terminal domain (LBD: S^71^-L^215^, α4-α9). The DBD consists of all necessary structural elements for DNA binding and the LBD consists of a well defined ligand binding cavity and also, it serves as the dimerization domain. The monomer is dumbbell shaped and the two major domains are connected by a small linker region. Two monomers in the asymmetric unit are packed together very tightly and two C-terminal helices of each monomer interact extensively to stabilize the homodimer. The overall architecture of Fad35R monomer structure is in good agreement with the structures of TetR [13], QacR [34], EthR [35] and CmeR [36]. However, closer analyses showed that two molecules of the crystallographic dimer are structurally asymmetric in nature and one of the DBD is twisted about the two-fold non-crystallographic axis (**Fig 1C**).

### N-terminal DNA binding domain

The DBD domain is composed of first ~ 70 residues, but the N-terminal 24 residues are disordered. The remaining ~46 residues, from A^24^ to R^70^, fold into three α-helices (α1, α2 and α3) connected by loops. The two DNA recognition helices, α2 and α3 form helix-turn-helix (HTH) motif which pack against the long N-terminal helix, α1 for stabilization. The sequence and structure alignment with other members of TTR family shows that many of the DNA binding residues are conserved (L^31^, A^34^, A^54^, Y^63^, R^64^, and H^65^). However, the DNA recognition helix, α3 of Fad35R is more solvent exposed and exhibits conformational flexibility, as evident from the lack of electron density for the side chains of predicted DNA binding residues, Y^63^ and R^64^ (**Fig 1D**). The Y^63^ is the most highly conserved DNA binding residue across the TTR family and distance between two Cα-atoms of Y^63^ and Y^63’^ of two monomers has been used to interpret DNA binding modes of the protein [12,17]. Analyses of ligand and DNA free crystal structures of TTR showed that distances between two conserved DNA binding tyrosines of two monomers vary between ~31 to 73 Å, suggesting that DNA binding domains are more flexible in the apo-state [12,17,35,36]. Two conserved tyrosines of Fad35R in the regular dimer, Y^63^ and Y^63’^ are distanced at ~ 48 Å Since the distance between two adjacent major groves in the B-form DNA is only ~ 34 Å, the disposition of two DBDs in the crystal structure of Fad35R suggest that two DBDs adopt an open conformation that mimics one of the “de-repressed” state like conformations, accessible to apo-states. Analyses of DNA-bound structures of TTR members indicate that conformational freedoms of DBDs are restricted and two the recognition helices α3 and α3’ are separated by an average gap of 32 to 38 Å [22–26]. Distance between two recognition helices of Fad35R is larger than largest distance observed in the DNA bound mode; the apo-state of Fad35R does not represent a DNA binding competent conformation. Apo structures of SimR and SCO0520 regulators also display a non-DNA binding type of conformations [17,35].

### The C-terminal ligand binding and dimerization domain

**Fig 2:**
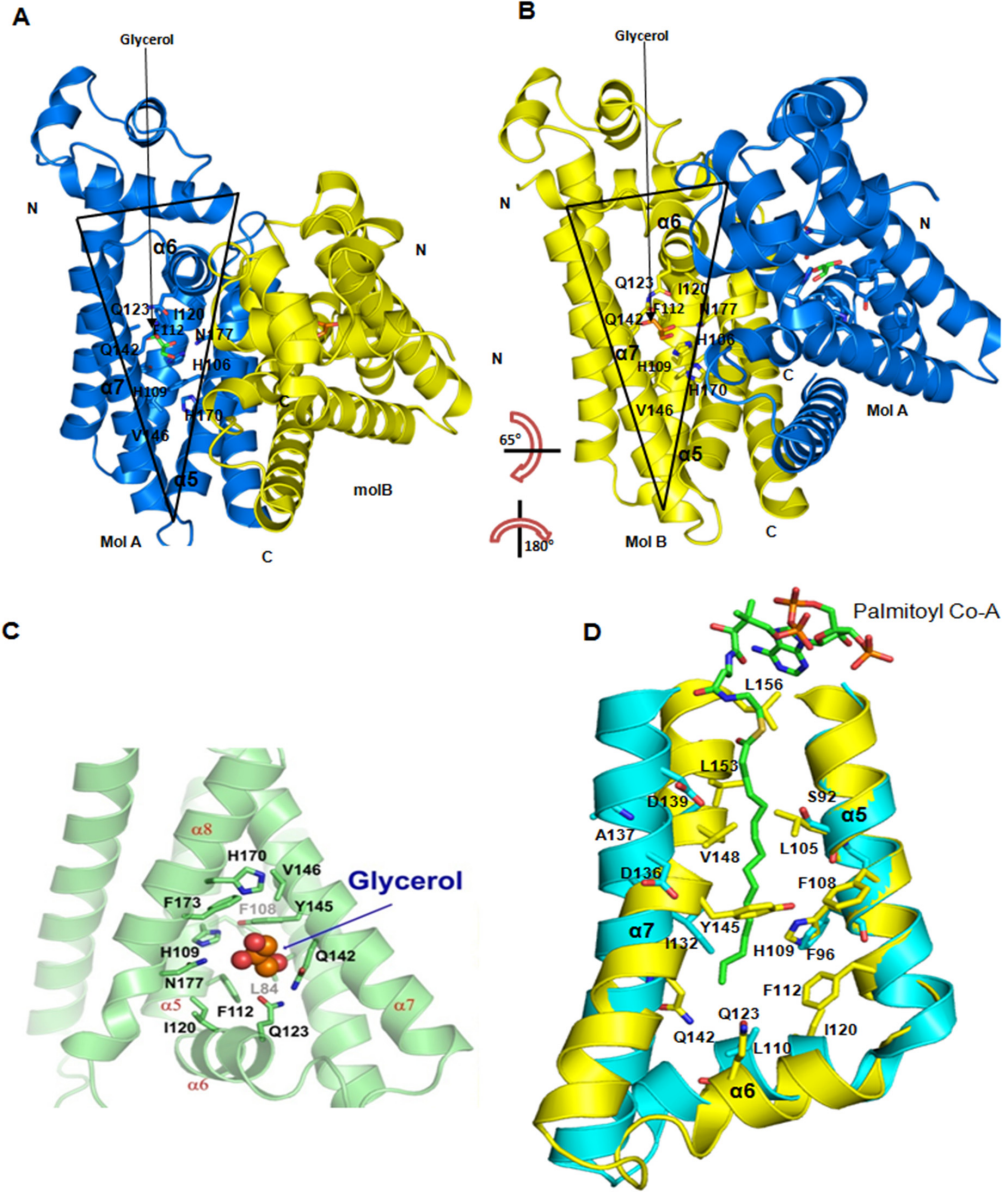
Structural view of ligand binding pocket. A & B) The open and opposite orientations of two triangular ligand binding cavities of two monomers (blue and yellow), superimposed by 180° rotation around vertical axis followed by 65° rotation of x-axis. C) The structural view of ligand binding pocket of one monomer. The glycerol is shown as space filling and predicted ligand binding residues are shown in sticks. D) View of Fad35R (yellow) ligand binding pocket superimposed to that of DesT (cyan) bound to palmitoyl Co-A (PCA) (shown as sticks, orange) indicate that fatty acid tail can fit well into the cavity and L140, I132, and S92 may form interactions with PCA.

The C-terminal domain is formed of residues, 71–215 and it is composed of six major helices, α4 to α9, which encompass the ligand binding site and also provide the dimerization interface. The fourth helix, α4 which extends from the base of DBD, runs parallel to α7. The seventh helix is usually kinked in most TTRs, but it adopts a straight conformation in Fad35R. The straight conformation enables α7 to mediate multiple contacts with α4 and also, the absence of curvature creates a large ligand binding cavity within the LBD. The triangular ligand binding cavity is formed by helices α5, α6, and α7 in each monomer and ttwo cavities are solvent accessible but face opposite sides of the dimer. A rotation of 180° along the y-axis, parallel to the two-fold NCS axis, followed by rotation around x-axis by 65°, superposes the ligand binding cavity of molA onto molB (**Fig 2A and 2B**). Structural dispositions of the two ligand binding cavities at opposite sides of the dimer would facilitate ligand access.

Although the Fad35R was crystallized in the apo-state with no ligands added before crystallization, we noticed an unaccounted electron density within the ligand binding cavity. We modeled a glycerol molecule into this unaccounted electron density and refined it. The binding pocket is hydrophobic and lined with several hydrophobic and aromatic residues L^84^, F^108^, F^112^, I^120^, Y^145^, V^146^, and F^173^, suitable for binding fatty acid tails. The main residues that participate in hydrogen bonding with ligands are H^109^, F^112^, I^120^, Q^123^, Q^142^, Y^145^, V^146^, H^170^, F^173^, and N^177^ (**Fig 2C**). The ligand binding cavity is positively charged, consistent with the presence of histidines and glutamines in the binding cavity. Superposition with crystal structure of DesT bound to DNA shows that fatty acid will bind through induced-fit mechanism because the straight α7 would block the binding pocket of fatty acid tail. In order to accommodate its tail, the incoming fatty acid should induce conformational changes that would result in the movement of α7 out of fatty acid tail binding pocket (**Fig 2D**). Further, alignment of residues within the ligand binding tunnel clearly shows that fatty acid tail would mediate several hydrophobic interactions with these residues. Superposition of Fad35R on to ligand bound structures of QacR and EthR revealed that the triangular cavity is lined with an array of residues that can mediate hydrogen bonding with other ligands such antibiotics and citrate [37,38].

### Crystal packing analyses reveals alternative dimer interface

**Fig 3:**
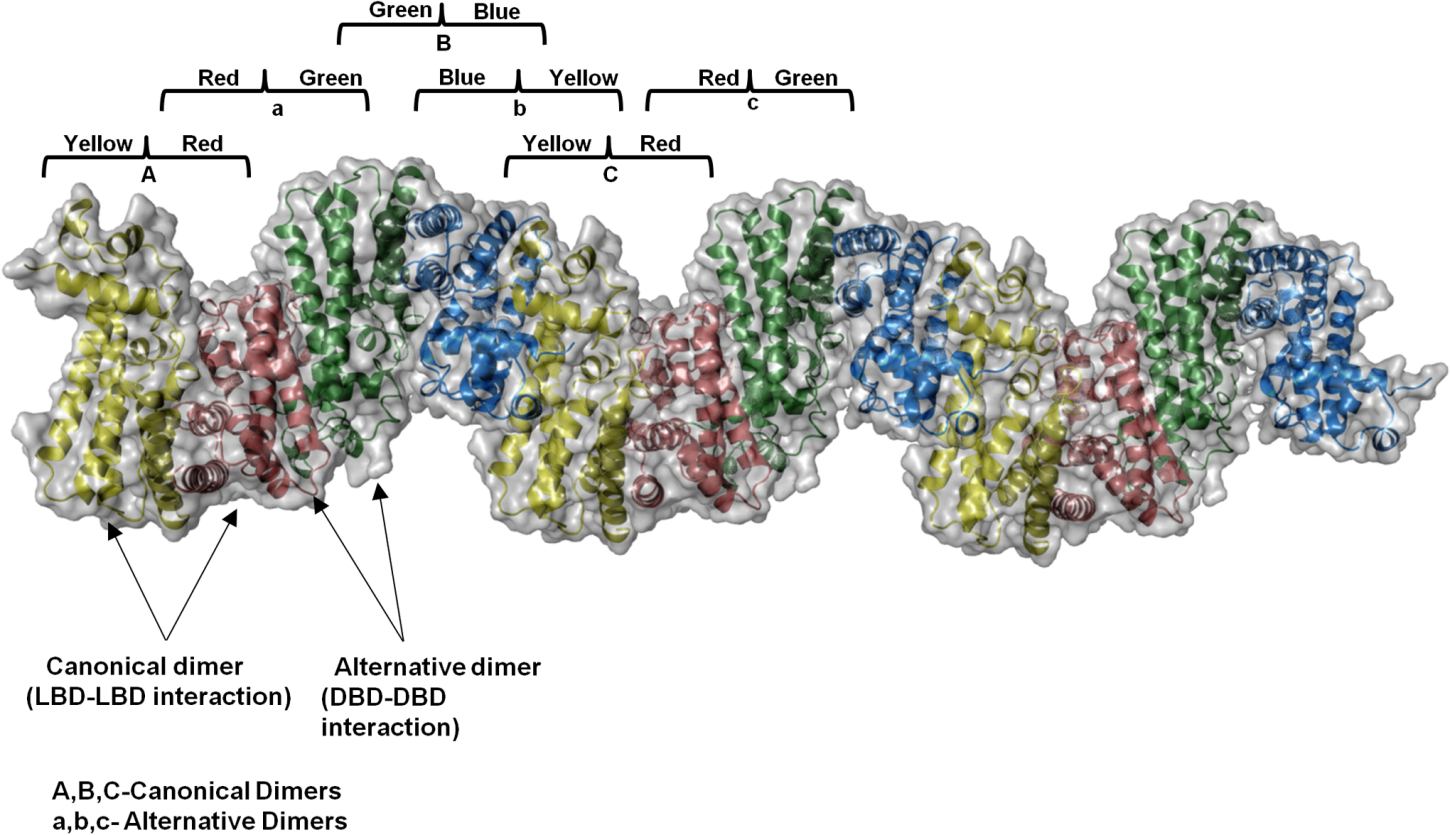
Crystal packing representations of canonical and alternative dimers. View of repeating alternate quaternary states; alternate arrangement of two canonical dimers (yellow-red, labeled as "A" and green-blue labeled as "B") and alternative dimers (red-green, labeled as "a" and blue-yellow, labeled as "c" repeat along the y-axis and form a tetrameric assembly).

Structural comparison with DesT and CgmR shows that the distance between the helices α3 and α3’ of Fad35R is more due to asymmetrical disposition of DBD of the chain B with respect to chain A DBD. Another consequence of asymmetric packing of two DBDs is the formation of alternative dimer interface. Analyses of crystal packing suggest that the alternative dimer interface is formed between DBDs of the canonical dimer with that of adjacent symmetry mates (**Fig 3**). The canonical and alternative dimers can be generated repeatedly along the y-axis by applying the crystallographic symmetry operations in the *P*2_1_2_1_2_1_ space group. The alternative dimer is formed by applying the symmetry operation-x+1/2, y+1, z to molA or x-1/2, y-1, z to molB of the Fad35R homodimer. In addition, evaluation of crystal packing shows that a continuous high-order oligomeric array could be generated along the y-axis and interfaces of higher-order assembly can be described by these two types of dimeric interfaces (**S1 Fig**). The alternative dimeric interface may also coincide with the tetramerization interface between two canonical dimers, suggesting the possibility of tetramerization. The Superamolecular structure can also be adequately described by repeating tetrameric assembly which has two canonical dimers connected through the alternative dimeric interface.

### Canonical dimer interface

**Fig 4:**
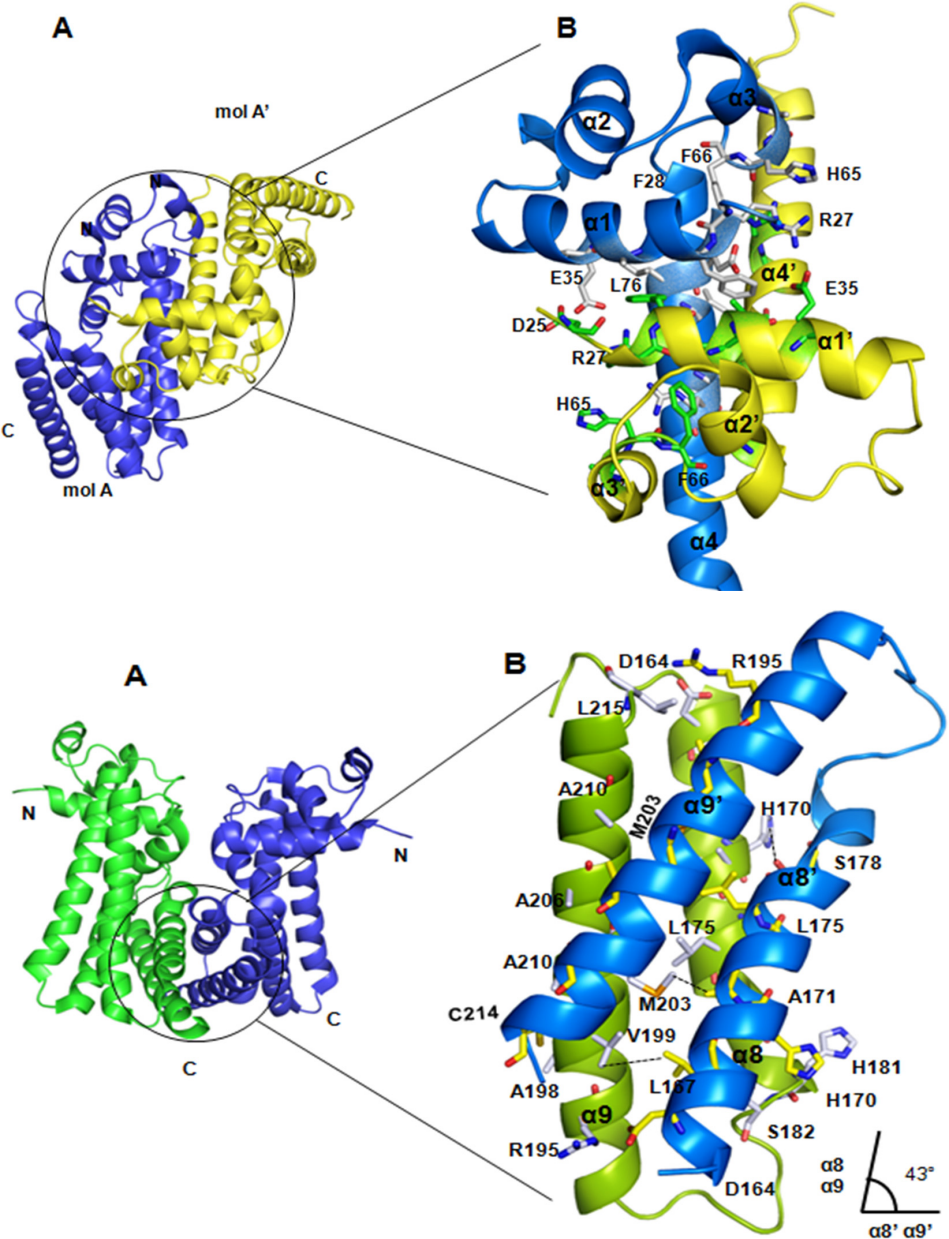
Structural view of dimerization interfaces. A) The canonical dimer (blue and green) found in the asymmetric unit. Two dimerization helices (α8, α9 and α8’, α9’) that form four helix bundle at the interface are shown. B) A close-up view of dimerization interface (cartoon) and important residues that make interactions are shown in sticks. The crossing angle between dimerization units is shown below. C) The alternative dimer formed between one monomer of the canonical dimer (molA, cyan) and one monomer of the symmetry mate (mol A', green). D) The dimerization interface of alternative dimer shows alternative dimerization interface is contributed by two DBDs (α1 and α4).

The last two helices, α8 and α9 from each monomer of Fad35R are the principle dimerization units which interact to form a four helix bundle (α8, α8' and α9, α9') at the dimerization interface. We refer to this dimer as the “canonical dimer” since this type of dimerization is observed in most of the TTR members. A total of ~ 4 hydrogen bonds and ~ 7 salt bridges stabilize the subunit-subunit interactions and give rise to an extensive dimer interface which buries a solvent accessible surface area of ~ 2330 Å^2^, which is 12.5% of the total solvent accessible surfaces of one subunit (**S1 Table**). The dimerization unit of monomer A, α8 and α9, cross the dimerization unit of the monomer B (α8' and α9') at ~ 43 Å (**Fig 4A and 4B)**. This criss-cross arrangement forces these two helix pairs to stack on top of each other, forming the four helix bundle and constraining the dispositions of two DBDs and LBDs as well. In this arrangement, DBD of one of the monomer swings away from the two-fold NCS symmetry by 12.4 Å and therefore, the two DNA binding helices are spaced at ~ 48 Å. In addition, the outward movement of one DBD positions it in close proximity to the DBD of its adjacent symmetry mate in the crystal lattice. This proximity creates an alternative DBD-DBD dimerization interface between each monomer of the canonical dimer and their respective symmetry mates. Criss-cross packing of the canonical dimer also positions two ligand binding pockets at the opposite sites of the dimer.

### Alternative dimer interface

The α1 of the DBD and α4 of LBD contribute majorly to the alternative dimer interface. In contrast to canonical dimeric interface, which is contributed mainly by two LBDs (α8 and α9), alternative dimer interface is stabilized by both DBD and LBD. The first helices of two DBDs, α1 and α1’ contribute four main salt bridges (R^27^ and R^83^ make two each with E^35^ and E^75^ respectively) and other interactions to provide stability to the alternative dimer. Also, the first helices of LBDs, α4 and α4’ participate in the alternative dimer formation and further strengthen the interactions between two monomers. This DBD-DBD dimer interface displays a number of specific interactions and buries a large solvent accessible surface area. In the alternative dimer, the subunit interface buries approximately 2710 Å^2^ (15%) of the total solvent accessible surface area of about 18240 Å^2^ per subunit. The residues R^27^, E^35^, R^64^, E^75^, R^83^, D^111^, E^116^ and E^135^ from the helices α1, α3, α4, α5 and α7 from both the monomers mediate specific interactions that stabilize the alternative homodimer (**Fig 4C and 4D and S2 Table)**. The estimated buried solvent accessible surface area of the alternative dimer is in agreement with the mean distribution of values calculated for homodimeric structures in the PDB. Interestingly, the alternative dimeric interface also coincides with a potential tetramerization interface that connects two canonical dimers.

The two recognition helices of the alternative dimer are distanced at ~35 Å, which is equal to the distance corresponding to the separation of two consecutive major groves in DNA. However, using our structure, it is not possible to predict whether the observed alternative dimer would recognize DNA. The two primary recognition helices, α3 and α3^'^ of alternative dimer are well exposed to solvent, suggesting that the alternative dimer may be able to bind DNA. We investigated the possibility whether alternative dimer mimics the DNA bound "repressed state". We compared the structure of alternative dimer with DNA bound structures of SimR and QacR [15,16]. Alternative dimer of Fad35R shows no or minimal structural similarity due to unusual packing interactions involving α4 and α1. Therefore, we conclude that the alternative dimer cannot be considered as a "repressed" state of Fad35R, but it may represent one of the "closed-state" forms in which both recognition helices are closer in space and the ligand binding pockets are inaccessible. Two HTH motifs of Fad35R come much closer in the alternative dimer, similar to that of in the DNA-bound structures of other TTRs, but they adopt different conformation and ligand binding pockets are closed.

### Higher-order oligomerization of Fad35R

**Fig 5:**
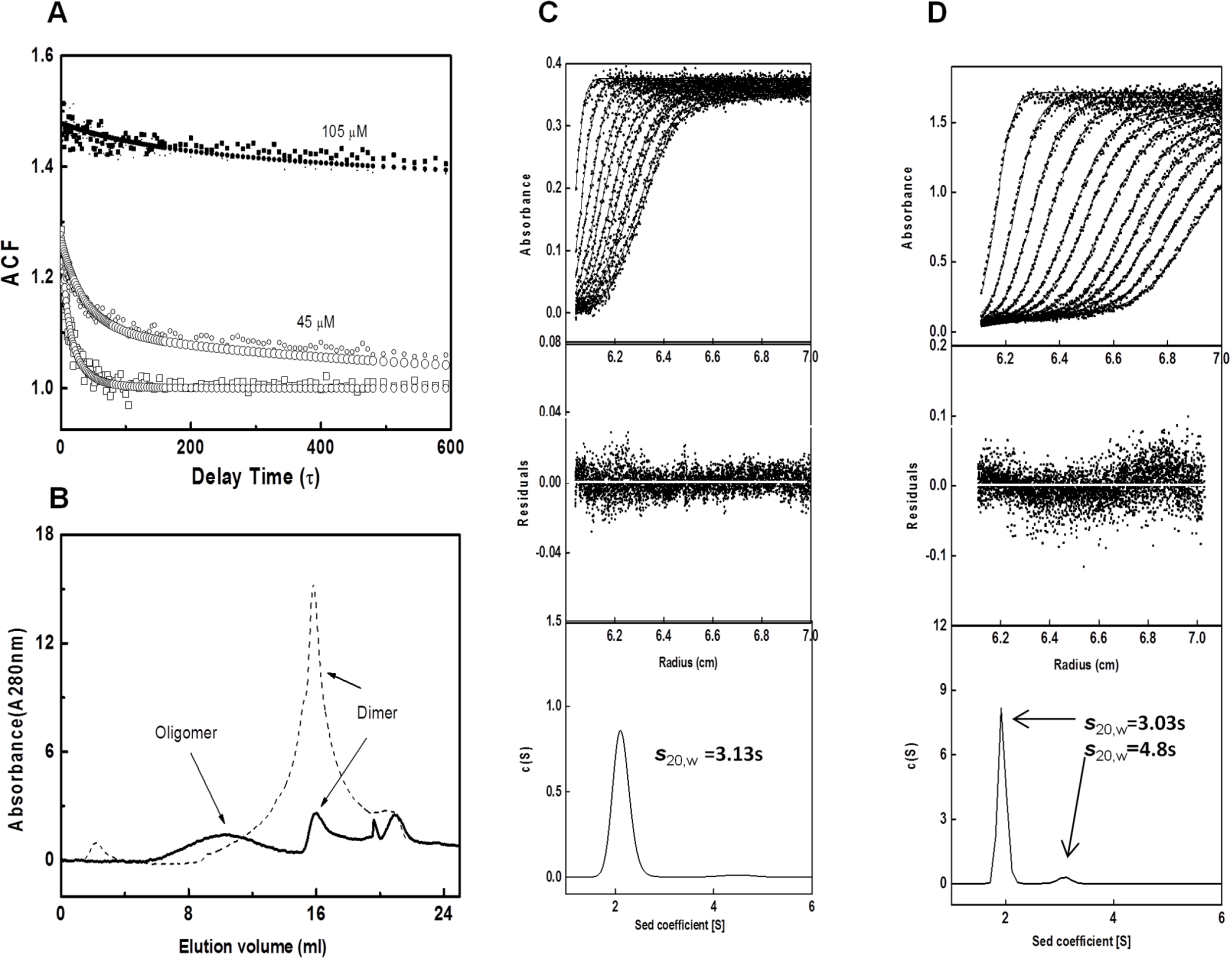
Ligand induced oligomerization. A) Particle size analyses shows that Fad35R (oligomerizes in the presence of tetracycline. Raw data (no tetracycline-□, 45 μM tetracycline-○, and 100 μM tetracycline-■) are fit to single exponential decay model. B) Size exclusion analyses of Fad35R oligomerization, plotted as elution volume versus absorbance, in the absence (dash) and presence of 100 μM tetracycline (dark). C & D) Sedimentation velocity profiles and analyses of Fad35R in the absence and presence of tetracycline at indicated concentrations. The S_20,W_ and respective molecular weights are shown.

Since Fad35R behaves as homodimer in solution, we examined its assembly state in the presence of tetracycline (Tc), a known inducer [10]. We analyzed the assembly state of Fad35R as a function of Tc concentration using size-exclusion, particle size analysis (light scattering), and sedimentation approaches. Addition of Tc to solution containing Fad35R causes changes in the intensity profiles and subsequent changes in the decay of autocorrelation function. A single exponential function is not adequate to fit the normalized autocorrelation function as it does not describe the data well (**Fig 5A**). As shown by the fit, data collected in the presence of Tc deviate from the model, suggesting that a more complex model is needed to describe the data adequately. The deviation from the model indicates the presence of mixed oligomeric Fad35R populations. Further, the average particle size estimated increases upon Tc addition suggesting that Tc bound Fad35R has the propensity to form higher oligomers (**S3 Table)**. Next, we examined the effect of Tc on Fad35R oligomerization using size-exclusion chromatography as another independent technique. Purified Fad35R and mixture of Fad35R-Tc in a molar ratio of 1:2 were loaded onto analytical gel filtration column under similar buffer conditions. Elution profiles of Fad35R and Fad35R-Tc mixture are shown in **Fig 5B**. Fad35R elutes as homodimer as expected, the Tc bound Fad35R, however, elutes as broad peak indicating the oligomerization as well as heterogeneity of the sample.

Although higher-order oligomerization of Fad35R was observed in the presence of tetracycline, the state of oligomers could not be well established. Therefore, we performed sedimentation velocity experiments and monitored the movement of Fad35R in the absence and presence of limited amount of tetracycline to prevent aggregation. Analyses of profiles of purified Fad35R protein shows a sharp peak with sedimentation coefficient, S_20,w_ ~3.13, which is expected for a protein with molecular weight of ~ 48 kDa, confirming that Fad35R exists as a homodimer in solution (**Fig 5C**). Next, we incubated Fad35R (30 μM) with 50 μM of tetracycline for 4 hours and repeated the experiments. Analyses of sedimentation profile showed that although the major fraction appeared to be of homodimer and a minor fraction with S_20,w_ ~ 4.82, corresponding to the molecular weight of ~ 94 kDa was observed (2–4%) (**Fig 5D**). Sedimentation experiments at higher Tc concentrations could not be performed as the absorbance profiles increased dramatically and profiles had scattered appearance (**S2 Fig**). The scattered profiles may also indicate the heterogeneity of the sample due to the presence of multiple species present in the solution. Therefore, we conclude that Fad35R exists predominantly as homodimer although with propensity to tetramerize and oligomerize in the presence of Tc.

## Discussion

This study presents the first crystal structure of a FadR homologue, Fad35R from mycobacterium. The structure of Fad35R shows regular α-helical fold, typical signature of TTR family, but it exhibits several distinct structural features. Fad35R was crystallized as a homodimer in the asymmetric unit, with each subunit consisting of one N-terminal HTH motif and a C-terminal LBD. Some of the major structural features that are unique to Fad35R structure are: straightening of canonical helix, α7 which is kinked in most of the TTR members; the four helix bundle formed with the crossing angle of ~ 43° between dimerization units (α8-α9 and α8'-α9') of the two monomers; two large, triangular ligand binding cavities that face opposite sides of the canonical dimer; asymmetrical disposition of two HTH motifs in the apo-state state; and formation of alternative dimeric interface or potential tetramerization interface. These tertiary and quaternary structural properties of Fad35R may likely to reflect on biochemical properties of Fad35R.

The crystal structure of Fad35R is in its free state and the distance between two recognition helices are ~ 48 Å, which is much larger than the distance between two consecutive major groves of the DNA [12,13]. This indicates that two DBDs of Fad35R assume a conformation which is not compatible with that of DNA binding. It is known that ligand binding traps two DNA binding HTH motifs in to a conformation that would prevent the protein from binding to DNA. Crystal structures of TTR members bound to DNA show that two DBDs are separated by not more than 38 Å. On the contrary, binding of effector ligands to LBD increase the distance between two DBDs to more than 38 Å and trap the regulator in the derepressed state [17,35–42]. Many TTR family proteins crystallize in apo form with their DNA binding helices of two monomers well separated, a conformation not consistent with the DNA binding form [17,35,36]. Comparison of Fad35R structure with apo forms of other TTR members show that apo forms of TTR members exhibit structural heterogeneity to a great extent [12]. For example, superpositioning shows that N-terminal DBDs of Fad35R and DhaS superpose well but C-terminal domain of DhaS is more open due to short helix 4, α4 [40]. Although Fad35R is not bound to any inducer molecule, the two DBDs assume a more open conformation that does not favor DNA binding. This is consistent with earlier observations that apo-TTR proteins do not crystallize in their DNA bound form [12,13]. Although the possibility exists that crystal packing may have promoted non-DNA binding conformation in the Fad35R structure, the observation that other TTR members like DhaS, SCO052, AcrR, and SimR also crystallize with their two DBDs separated by a more than 38 Å suggests that DBDs of TTR family exhibit conformational flexibility [12,15,17,35,36,40]. The ligand binding cavities are in open-state and fatty acid tails are expected to be buried inside the cavity. The extensive tunnel like architecture, housing a mixture of hydrophobic and polar residues explains that both hydrophobic and polar ligands can bind. Superposition with ligand bound structures of DesT and QacR shows that fatty acids would bind to this cavity with their hydrophobic tails inserted whereas tetracycline or citrate intercalate within the triangular cavity. Structural comparisons with ligand bound structures of DesT and QacR also suggest that the straight helix, α7 may serve as the ligand sensitive structural element and binding of either fatty acid or tetracycline will induce a kink to helix7 [18,37]. We also predict that the bending of helix7 would displace the dimerization helices α8 and α9, resulting in the rotation about dimerization axis as proposed earlier [35]. These ligand mediated conformational changes at the LBD would re-position the DNA binding domains, leading to the change in the functional status of the protein.

The open arrangement of two DBDs and well exposed ligand binding pockets are two of many unique features of the Fad35R structure. Two dimerization units of each monomer interact at an angle of ~ 43°, which is a relatively low angle of crossing as compared to that of other members. Analyses of cross over angles of dimerization in this family members reveal that two main dimerization of helices cross each other at an angle of 65°-80° [13–17, 35–42]. The cross-over angles change when ligand binding induces structural changes. For example, ligand binding rotates one subunit of SimR by 16°, relative to the other. This rotation results in the decrease of cross over angle and separates the two DBDs further, favoring the derepressed state like conformation [35]. We assume that unusual low cross-over angle between two dimerization units may have manifested asymmetric dimerization and also would have favored the alternative dimer/tetramerization interface. Another possibility is that crystal packing forces would have favored asymmetric canonical dimerization and formation of alternative dimers. We analyzed crystal contacts and found that both monomers in the canonical dimer have equal contact energies as revealed by similar type of intermolecular interactions with their symmetry mates. Interestingly, both dimerization interfaces bury significant and similar amount of solvent accessible surface areas. These observations lead us to propose that both type of dimers may likely to be in equilibrium, although fraction of alternative dimer in solution may be low due to non-specific contacts between two monomers in the alternative dimer. Crystal structures of Fad35R in complex with DNA or inducer are needed to evaluate the correlation between subunit rotation and functional states of Fad35R.

The general derepression mechanism of TTR family involves ligand induced allosteric conformational changes which reposition two DBDs, resulting in the separation of two recognition helices to a configuration that would reduce the affinity for the DNA. Although deviations from this mechanism have not been observed, structural studies revealed that the mode of DNA recognition is dictated by unique conformational properties of the regulators [13–17]. DesT uses phosphate-backbone contacts for recognition in contrast to TetR, which uses DNA base specific hydrogen bonds [13,18]. SimR uses a 28-residue, positively charged N-terminal extension that binds to the minor grove of DNA for additional stability [15]. DNA binding was shown to stabilize the conformation of N-terminal extension of SimR. EthR oligomerizes on the DNA to form octameric state and similarly, QacR binds cooperatively to its DNA as dimer of dimmers [16,37]. Fad35R also has 25-residue N-terminal extension like SimR, binds to long chain fatty acids like DesT, and also exhibits oligomerization tendency like EthR and QacR [15,18,41]. Fad35R displays a mixture of structural and functional features of different regulators. The alternative quaternary structure has not been reported for TTR or GntR family regulators. BenM, a LysR-type transcriptional regulator (LTTR) forms infinite oligomeric arrays in crystals [42]. The study proposed a general oligomerization scheme for LTTR family but biological significance of oligomerization was not proposed. The higher-order oligomeric array displayed by Fad35R in the crystal packing is consistent with earlier observation in the BenM structure. However, it is not clear from this work or previous work about the significance of propensity to form higher oligomers by BenM and Fad35R. In summary, unique tertiary and quaternary structural properties of Fad35R, its propensity to form oligomers, and ability to recognize chemically diverse ligands suggests that a complex repression and derepression mechanism for Fad35R. Structures of Fad35R in complex with cognate ligands will further enhance our understanding on regulatory mechanisms of TTR family in mycobacterium.

## Supporting Information

S1 Fig Crystal packing representations of Fad35R along crystallographic y-axis. Two monomers of canonical dimer (blue and yellow) are presented by capital letters A-F (on top) and two mononers of alternative dimers (blue and yellow) are represented by small letters a-f (on top). The repeated arrangement of canonical and alternative arrangement creates a superamolecular assembly on the y-axis. The respective dimerization interfaces are shown at the bottom.
(DOC)

S2 Fig Sedimentation velocity profiles of Fad35R in the presence of 100.0 μM tetracycline. The y-axis represents optical density of the sample, monitored at 280 nm and x-axis represents radius along the cell. Due to relatively high absorbance of tetracycline at 280 nm and sample heterogeneity induced by tetracycline, the absorbance profiles are scattered.
(DOC)

S1 Table Interactions that stabilize canonical dimer interface. (DOC)

S2 Table Interactions that stabilize alternative dimer interface. (DOC)

S3 Table Particle size analyses of Fad35R. (DOC)

## References

[1] Cole ST, Brosch R, Parkhill J, Garnier T, Churcher C, Haris D, et al Deciphering the biology of *Mycobacterium tuberculosis* from the complete genome sequence. Nature. 1998; 393: 537–544. 9634230 10.1038/31159

[2] Lee W, VanderVen BC, Fahey RJ, Russell DG. Intracellular *Mycobacterium tuberculosis* exploits host-derived fatty acids to limit metabolic stress. J Biol Chem. 2013; 288: 6788–6800. 10.1074/jbc.M112.445056 23306194 PMC3591590

[3] Gao LY, Laval F, Lawson EH, Groger RK, Woodruff A, Morisaki JH, et al Recruitment of KasB in *Mycobacterium* mycolic acid biosynthesis, cell wall impermeability and intracellular survival: implications for therapy. Mol Microbiol. 2003; 49: 1547–1563. 12950920 10.1046/j.1365-2958.2003.03667.x

[4] Kendall SL, Burgess P, Balhana R, Withers M, Ten Bokum A, Lott JS, et al Cholesterol utilization in mycobacteria is controlled by two TetR-type transcriptional regulators: kstR and kstR2. Microbiology. 2010; 156: 1362–1371. 10.1099/mic.0.034538-0 20167624 PMC3068626

[5] Mondino S, Gago G, Gramajo H. Transcriptional regulation of fatty acid biosynthesis in mycobacteria. Mol Microbiol. 2013; 89: 372–387. 10.1111/mmi.12282 23721164 PMC3752660

[6] Blattner FR, Plunkett G 3rd, Bloch CA, Perna NT, Burland V, et al The complete genome sequence of *Escherichia coli* K-12. Science. 1997; 277: 1453–1462. 9278503 10.1126/science.277.5331.1453

[7] Haydon DJ, Guest JR. A new family of bacterial regulatory proteins. FEMS Microbiol Lett. 1991; 63: 291–295. 2060763 10.1016/0378-1097(91)90101-f

[8] Vindal V, Suma K, Ranjan A. GntR family of regulators in *Mycobacterium smegmatis*: a sequence and structure based characterization. BMC genomics. 2013; 8:289. 10.1186/1471-2164-8-289 PMC2018728 17714599

[9] Biswas RK, Dutta D, Tripathi A, Feng Y, Banerjee M, Singh BN. Identification and characterization of Rv0494: a fatty acid responsive protein of the GntR/FadR family from *Mycobacterium tuberculosis* . Microbiology 2013; 159: 913–923. 10.1099/mic.0.066654-0 23475950

[10] Anand S, Singh V, Singh AK, Mittal M, Datt M, Subramani GL, et al Equilibrium binding and kinetic characterization of putative tetracycline repressor family transcription regulator Fad35R from *Mycobacterium tuberculosis* . FEBS J. 2012; 279: 3214–3228. 10.1111/j.1742-4658.2012.08707.x 22805491

[11] Ramos JL, Martinez-Bueno M, Molina-Henares AJ, Teran W, Watanabe K, et al The TetR Family of Transcriptional Repressors. Microbiol Mol Biol Rev. 2005; 69: 326–356. 15944459 10.1128/MMBR.69.2.326-356.2005 PMC1197418

[12] Reichheld SE, Yu Z, Davidson AR. The induction of folding cooperativity by ligand binding drives the allosteric response of tetracycline repressor. Proc Natl Acad Sci USA. 2009; 106: 22263–22268. 10.1073/pnas.0911566106 20080791 PMC2799725

[13] Orth P, Schnappinger D, Hillen W, Saenger W, Hinrichs W. Structural basis of gene regulation by the tetracycline inducible Tet repressor-operator system. Nat Struct Biol. 2000; 7: 215–219. 10700280 10.1038/73324

[14] Itou H, Watanabe N, Yao M, Shirakihara Y, Tanaka I. Crystal Structures of the Multidrug Binding Repressor *Corynebacterium glutamicum* CgmR in Complex with Inducers and with an Operator. J Mol Biol. 2010; 403: 174–184. 10.1016/j.jmb.2010.07.042 20691702

[15] Le TB, Schumacher MA, Lawson DM, Brennan RG, Buttner MJ. The crystal structure of the TetR family transcriptional repressor SimR bound to DNA and the role of a flexible N-terminal extension in minor groove binding. Nucleic Acids Res. 2011; 39: 9433–9447. 10.1093/nar/gkr640 21835774 PMC3241653

[16] Schumacher MA, Miller MC, Grkovic S, Brown MH, Skurray RA, Bernnan RG. Structural basis for cooperative DNA binding by two dimers of the multidrug-binding protein QacR. EMBO J. 2002; 21: 1210–1218. 11867549 10.1093/emboj/21.5.1210 PMC125875

[17] Filippova EV, Chruszcz M, Cymborowski M, Gu J, Savchenko A, Edwards A, et al Crystal structure of a putative transcriptional regulator SCO0520 from *Streptomyces coelicolor* A3 (2) reveals an unusual dimer among TetR family proteins. J Struct Funct Genomics. 2011; 12:149–157. 10.1007/s10969-011-9112-4 21625866 PMC4413006

[18] Miller DJ, Zhang YM, Subramanian C, Rock CO, White SW. Structural basis for the transcriptional regulation of membrane lipid homeostasis. Nat Struct Mol Biol. 2010; 17: 971–975. 10.1038/nsmb.1847 20639888 PMC2935088

[19] Hendrickson WA, Horton JR, Le Master DM. Selenomethionyl Proteins Produced for Analysis by Multiwavelength Anomalous Diffraction (MAD): A Vehicle for Direct Determination of Three-Dimensional Structure. EMBO J. 1990; 9: 1665–1672. 2184035 10.1002/j.1460-2075.1990.tb08287.x PMC551863

[20] Otwinowski Z, Minor W. Processing of X-ray diffraction data collected in oscillation mode. Elsevier 1997; 276: 307–326. 10.1016/S0076-6879(97)76066-X 27754618

[21] Schneider TR, Sheldrick GM. Substructure solution with SHELXD. Acta Crystallogr D Biol Crystallogr. 2002; 58: 1772–1779. 12351820 10.1107/s0907444902011678

[22] Sheldrick GM. Experimental phasing with SHELXC/D/E: combining chain tracing with density modification. Acta Crystallogr D Biol Crystallogr. 2010; 66: 479–485. 10.1107/S0907444909038360 20383001 PMC2852312

[23] Pape T, Schneider TR. HKL2MAP: a graphical user interface for macromolecular phasing with SHELX programs. J Appl Crystallogr. 2004; 37: 843–844.

[24] Emsley P, Cowtan K. Coot: model-building tools for molecular graphics. Acta Crystallogr D Biol Crystallogr. 2004; 60: 2126–2132. 15572765 10.1107/S0907444904019158

[25] Murshudov GN, Vagin AA, Dodson EJ. Refinement of macromolecular structures by the maximum-likelihood method. Acta Crystallogr Biol Crystallogr. 1997; 53: 240–255. 15299926 10.1107/S0907444996012255

[26] McCoy AJ, Grosse-Kunstleve RW, Adams PD, Winn MD, Storoni LC, Read RJ. Phaser crystallographic software. J Appl Crystallogr. 2007; 40: 658–674. 19461840 10.1107/S0021889807021206 PMC2483472

[27] Read RJ. Improved Fourier coefficients for maps using phases from partial structures with errors. Acta Crystallogr A. 1986; 42: 140–149.

[28] Cowtan K. General quadratic functions in real and reciprocal space and their application to likelihood phasing. Acta Crystallogr D Biol Crystallogr. 2000; 56: 1612–1621. 11092927 10.1107/s0907444900013263

[29] Cowtan K. The Buccaneer software for automated model building. 1. Tracing protein chains. Acta Crystallogr D Biol Crystallogr. 2006; 62: 1002–1011. 16929101 10.1107/S0907444906022116

[30] Winn MD, Isupov MN, Murshudov GN. Use of TLS parameters to model anisotropic displacements in macromolecular refinement. Acta Crystallogr D Biol Crystallogr. 2001; 57: 122–133. 11134934 10.1107/s0907444900014736

[31] Laskowski RA, MacArthur MW, Moss DS, Thornton JM. PROCHECK: a program to check the stereochemical quality of protein structures. J Appl Crystallogr. 1993; 26: 283–291.

[32] Krissinel E. Crystal contacts as nature’s docking solutions. J Comput Chem. 1995; 31: 133–143. 10.1002/jcc.21303 19421996

[33] Hayes D, Laue T, Philo J. Program Sednterp: Sedimentation Interpretation Program, Alliance Protein Laboratories, Thousand Oaks, CA 1995.

[34] Schuck P. Size-distribution analysis of macromolecules by sedimentation velocity ultracentrifugation and lamm equation modeling. Biophys J. 2000; 78: 1606–1619. 10692345 10.1016/S0006-3495(00)76713-0 PMC1300758

[35] Le TB, Stevenson CE, Fiedler HP, Maxwell A, Lawson DM, Buttner MJ. Structures of the TetR-like simocyclinone efflux pump repressor, SimR, and the mechanism of ligand-mediated derepression. J Mol Biol. 2011; 408: 40–56. 10.1016/j.jmb.2011.02.035 21354180

[36] Li M, Gu R, Su CC, Routh MD, Harris KC, Jewell ES, et al Crystal structure of the transcriptional regulator AcrR from Escherichia coli. J Mol Biol. 2007; 374: 591–603. 17950313 10.1016/j.jmb.2007.09.064 PMC2254304

[37] Schumacher MA, Miller MC, Grkovic S, Brown MH, Skurray RA, Brennan RG. Structural mechanisms of QacR induction and multidrug recognition. Science. 2001; 294: 2158–2163. 11739955 10.1126/science.1066020

[38] Dover LG, Corsino PE, Daniels IR, Cocklin SL, Tatituri V, Besra GS, et al Crystal structure of the TetR/CamR family repressor *Mycobacterium tuberculosis* EthR implicated in ethionamide resistance. J Mol Biol. 2004; 340: 1095–1105. 15236969 10.1016/j.jmb.2004.06.003

[39] Gu R, Su CC, Shi F, Li M, McDermott G, Zang Q, et al Crystal Structure of the transcriptional regulator CmeR from *Campylobacter jejuni* . J Mol Biol. 2007; 372: 583–593. 17686491 10.1016/j.jmb.2007.06.072 PMC2104645

[40] Christen S, Srinivas A, Bahler P, Zeller A, Pridmore D, et al Regulation of the Dha operon of *Lactococcus lactis*: a deviation from the rule followed by the Tetr family of transcription regulators. J Biol Chem. 2006; 281: 23129–23137. 16760471 10.1074/jbc.M603486200

[41] Engohang-Ndong J, Baillat D, Aumercier M, Bellefontaine F, Besra GS, et al EthR, a repressor of the TetR/CamR family implicated in ethionamide resistance in mycobacteria, octamerizes cooperatively on its operator. Mol Microbiol. 2004; 51: 175–188. 14651620 10.1046/j.1365-2958.2003.03809.x

[42] Ezezika OC, Haddad S, Neidle EL, Momany C. Oligomerization of BenM, a LysR-type transcriptional regulator: structural basis for the aggregation of proteins in this family. Acta Crystallogr Sect F Struct Biol Cryst Commun. 2007; 63: 361–368. 17565172 10.1107/S1744309107019185 PMC2334995

