# Supplementary material for: Crystal Structure of Fad35R from Mycobacterium tuberculosis H37Rv in the Apo-State
Source: PLoS One. 2015 May 4;10(5):e0124333. doi: 10.1371/journal.pone.0124333 (PMC4418694; doi:10.1371/journal.pone.0124333)
Supplement: S1 Fig — Two monomers of canonical dimer (blue and yellow) are presented by capital letters A-F (on top) and two mononers of alternative dimers (blue and yellow) are represented by small letters a-f (on top). The repeated arrangement of canonical and alternative arrangement creates a superamolecular assembly on the y-axis. The respective dimerization interfaces are shown at the bottom. (DOC) [file pone.0124333.s001.doc]

**Figure S1: Crystal packing representations of Fad35R along crystallographic y-axis**

**Canonical dimer Non-canonical dimer**

**(LBD-LBD interaction)** **(DBD-DBD interaction)**

**A - F Canonical dimer**

**a - f Non-canonical dimer**
