# Supplementary material for: Crystal Structure of Fad35R from Mycobacterium tuberculosis H37Rv in the Apo-State
Source: PLoS One. 2015 May 4;10(5):e0124333. doi: 10.1371/journal.pone.0124333 (PMC4418694; doi:10.1371/journal.pone.0124333)
Supplement: S2 Fig — The y-axis represents optical density of the sample, monitored at 280 nm and x-axis represents radius along the cell. Due to relatively high absorbance of tetracycline at 280 nm and sample heterogeneity induced by tetracycline, the absorbance profiles are scattered. (DOC) [file pone.0124333.s002.doc]

**Figure S2: Sedimentation velocity profiles of Fad35R in the presence of 100.0 µM**
