# Supplementary material for: Crystal Structure of Fad35R from Mycobacterium tuberculosis H37Rv in the Apo-State
Source: PLoS One. 2015 May 4;10(5):e0124333. doi: 10.1371/journal.pone.0124333 (PMC4418694; doi:10.1371/journal.pone.0124333)
Supplement: S1 Table — (DOC) [file pone.0124333.s003.doc]

# Table S1 Interactions that stabilize canonical dimer interface

Chain Source atom Chain Target atom Distance (Å)

A 164(ASP)/OD1 B 195(ARG)/NH2 3.81

B 195(ARG)/NE 2.89

A 164(ASP)/OD2 B 195(ARG)/NH2 3.66

B 195(ARG)/NE 3.99

A 195(ARG)/NH2 B 164(ASP)/OD2 3.23

A 195(ARG)/NE B 164(ASP)/OD1 3.11

164(ASP)/OD2 3.57

A 164(ASP)/OD1 B 195(ARG)/NE 2.89

A 164(ARG)/OD2 B 195(ARG)/NH2 3.66

A 195(ARG)/NE B 164(ASP)/OD1 3.11

A 195(ARG)/NH2 B 164(ASP)/OD2 3.23
