# Supplementary material for: Crystal Structure of Fad35R from Mycobacterium tuberculosis H37Rv in the Apo-State
Source: PLoS One. 2015 May 4;10(5):e0124333. doi: 10.1371/journal.pone.0124333 (PMC4418694; doi:10.1371/journal.pone.0124333)
Supplement: S2 Table — (DOC) [file pone.0124333.s004.doc]

# Table S2 Interactions that stabilize alternative dimer interface

Chain Source atom Chain Target atom Distance (Å)

A 27(ARG)/NH2 B 35(GLU)/OE1 3.45

B 35(GLU)/OE2 2.46

A 27(ARG)/NE B 116(GLU)/OE2 3.81

A 35(GLU)/OE1 B 27(ARG)/NH2 2.82

A 65(HIS)/ND1 B 116(GLU)/OE1 2.81

A 65(HIS)/ND1 B 116(GLU)/OE2 3.50

A 75(GLU)/OE1 B 83(ARG)/NE 2.58

B 83(ARG)/NH2 3.70

A 83(ARG)/NE B 75(GLU)/OE1 2.78

A 83(ARG)/NH2 B 75(GLU)/OE1 3.75

A 116(GLU)/OE1 B 65(HIS)/ND1 3.26

A 116(GLU)/OE2 B 27(ARG)/NE 3.66

B 27(ARG)/NH1 3.00

B 65(HIS)/ND1 3.96

A 27(ARG)/NH2 B 35(GLU)/OE2 2.46

A 27(ARG)/NH1 B 115(GLY)/O 3.06

A 35(GLU)/OE1 B 27(ARG)/NH2 2.82

A 65(HIS)/O B 83(ARG)/NH1 3.03

A 75(GLU)/OE1 B 83(ARG)/NE 2.58

A 83(ARG)/NH1 B 65(HIS)/O 3.17

A 83(ARG)/NE B 75(GLU)/OE1 2.78

A 116(GLU)/OE2 B 27(ARG)/NH1 3.00
