# Supplementary material for: Crystal Structure of Fad35R from Mycobacterium tuberculosis H37Rv in the Apo-State
Source: PLoS One. 2015 May 4;10(5):e0124333. doi: 10.1371/journal.pone.0124333 (PMC4418694; doi:10.1371/journal.pone.0124333)
Supplement: S3 Table — (DOC) [file pone.0124333.s005.doc]

**Table S3. Particle size analyses of Fad35R**

| **Tetracycline Conc (mM)** | **Diffusion coefficient (cm^2^/s)** | **Diameter (Å)** |
| --- | --- | --- |
| **0** | 2.7E-7 | 1.8 |
| **0.002**  **0.011**  **0.022**  **0.5**  **1.05** | 2.8E-7  4.0E-8  1.1E-8  4.7E-9  4.0E-9 | 1.7  13.0  44.8  106.2  122.1 |
